# 3D-printed snorkel mask adapter for failed N95 fit tests and personal protective equipment shortages

**DOI:** 10.2217/3dp-2020-0018

**Published:** 2021-02-10

**Authors:** Shiv Dalla, Rohit Shinde, Jack Ayres, Stephen Waller, Jay Nachtigal

**Affiliations:** 1 ^1^University of Kansas School of Medicine, Kansas City, KS 66160, USA; 2 ^2^Unaffiliated, Philadelphia, PA 19104, USA

**Keywords:** 3D printers, 3D printing, COVID-19, infection control, N95, personal protective equipment, PPE, safety, stopgap PPE

## Abstract

Personal protective equipment (PPE) shortages persist amidst increasing COVID-19 caseloads. These shortages encouraged some to pursue 3D printing to produce stopgap N95 alternatives. The design presented is an adapter for a commercially available snorkel mask to serve as a full-face respirator, used in dire PPE shortages or in individuals who failed fit testing. Masks were fit tested at The University of Kansas Health System in Kansas City, KS. The mask was fit tested on 22 individuals who previously failed fit testing, and all passed qualitative fit testing with the snorkel mask, adapter and viral filter apparatus. The authors endorse this design as a stopgap measure, proven to be effective in situations of dire PPE shortage or for individuals who have failed fit testing with conventional PPE.

## Background

In December of 2019 the first cases of a novel coronavirus, named SARS-CoV-2, were reported in China [1]. As the virus spread across the continent, it proved to be both highly contagious and highly virulent [1]. Some infected patients were suffering from acute respiratory distress syndrome (ARDS) [1]. By the middle of March 2020, the World Health Organization had designated the COVID-19 outbreak as a pandemic. Reports of not only strained economies but also strained global healthcare systems flooded the news [2]. Within healthcare settings, the focus shifted to the adequacy of personal protective equipment (PPE) [3].

### PPE shortages

The shortage of PPE across the country has been widely discussed by government officials and healthcare practitioners as well as the general population [3–6]. Unfortunately, recent reports indicate the supply of PPE “*is running low again as the virus resumes its rapid spread and the number of hospitalized patients climbs”* [7]. Although the situation has improved since it was first evaluated earlier in the year, many experts predict that shortages persist [8], particularly in light of structural weaknesses which have yet to be resolved [9]. Additionally, some predict that it may take years in order for stockpiles to fully replenish, an increasingly urgent challenge as cases continue to rise across USA [8]. Shortages of PPE have repeatedly been shown to contribute to the spread of the virus, and *“the situation is especially dire at hospitals serving communities of color or patients on Medicaid”* [10]. PPE shortages have even been shown to disproportionately affect women. As most PPE is designed for men, women are often left with ill-fitting masks, goggles and gloves [11].

### Fit testing

In addition to PPE shortages, various studies have commented on difficulties regarding inadequate mask fitting [12,13]. Mask fit is an important consideration of mask efficacy and is typically measured by either qualitative fit testing (QLFT) or quantitative fit testing (QNFT) [12]. In QLFT, mask wearers are tested to see if they can detect bitter or sweet scents aerosolized around the masked wearer, whereas QNFT measures ratios of ambient aerosols inside and outside of the mask [12]. A 2018 Korean study reported QNFT pass rates of the four most common N95 models to be below 50%, with poorer fit test results observed for women compared with men [13]. The inherent challenge of the possibility of a poor mask fit is magnified during pandemic settings due to time pressures and limited availability of options.

### 3D-printed alternatives

As the aforementioned issues with PPE were playing out, communities of 3D printing aficionados, colloquially known as ‘makers,’ began to discuss the issue and created homemade masks, face shields and gowns. Specifically, those with access to 3D printers were encouraged to continually run their machines to produce face-shields and masks that could be used in the healthcare sector. The lack of adequate access to conventional N95 masks pushed for some to pursue 3D print and locally distribute masks to assist in reducing PPE shortages. The widespread availability of 3D printers and cost-effective nature of the manufacturing process makes it excellent for such purposes.

The design presented is an adapter that can be used with a commercially available snorkel mask in order to serve as a full face respirator in either the case of a PPE shortage, or more pertinently for those who are unable to pass fit testing with the available N95 respirators at their respective facilities. This article focuses on the design, manufacture and validation of the snorkel mask adapter design and its usage in the COVID-19 pandemic as well as future usage as stopgap PPE.

## Methods

### Adapter design

**Figure 1.**
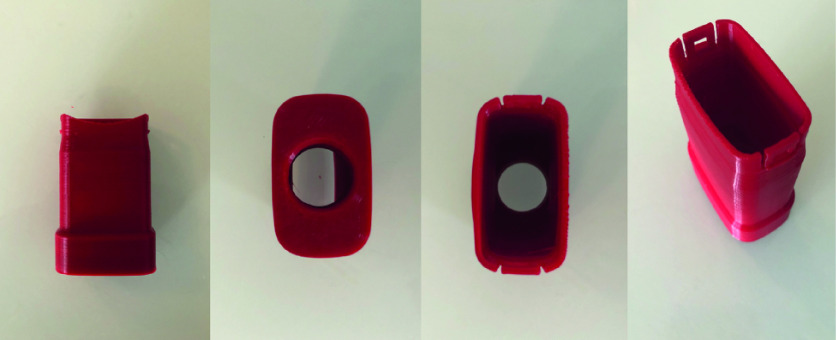
Multiple different views of a 3D-printed snorkel mask adapter produced with the specifications outlined in this manuscript.

**Figure 2.**
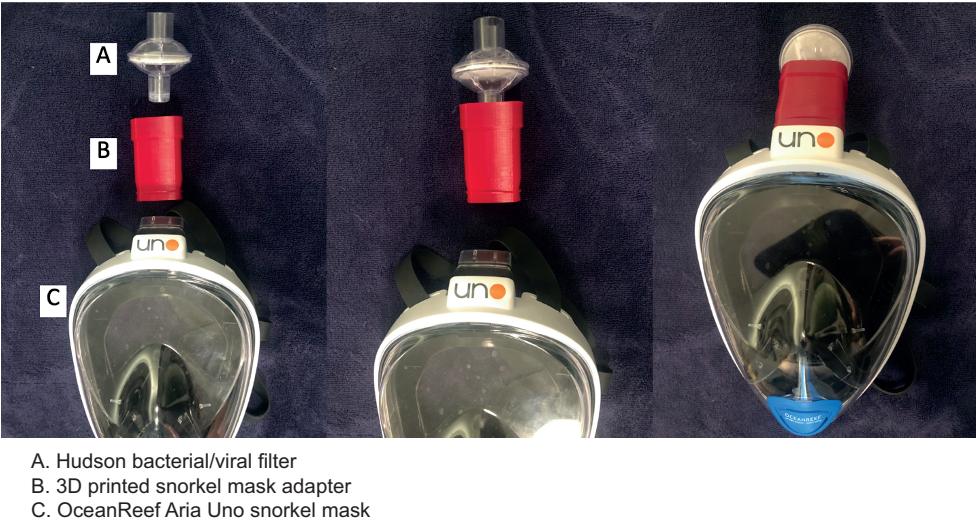
The stepwise assembly sequence of an OceanReef Aria Uno snorkel mask coupled to a Hudson bacterial/viral filter with the 3D-printed adapter.

The adapter was designed to connect an OceanReef Aria Uno [14] snorkel mask to a Hudson RCI In-Line Bacterial/Viral Filter [15] commonly found in both operating rooms and intensive care units for filtration and humidification of air in mechanically ventilated patients. These filters were chosen for their cost-effective nature, high availability during PPE shortages and low resistance to gas flow while maintaining 99.99% filtration efficiency [15]. Though prototyped and tested with the OceanReef Aria Uno [14], the adapter is compatible with all OceanReef snorkel masks as they all share the same snorkel connector to which the adapter attaches. The 3D printed adapter is shown in Figure 1 and the stepwise assembly sequence is shown in Figure 2.

### Design & 3D printing

This design was generated using commercial CAD software (SolidWorks) and exported to a Standard Tessellation Language (STL) file for compatibility with hobbyist 3D printer workflows. These workflows use a ‘slicer’ software to convert the universal STL file into G-code that is specific to an individual 3D printer. Two combinations of slicer and 3D printer were tested: the Cura for LulzBot slicer (version 3.6.18) was used to prepare G-code for a Lulzbot Taz5, and PrusaSlicer (version 2.3) was used to prepare G-code for a Creality CR-10S. The Lulbot Taz5 was outfitted with a Lulzbot 0.5 mm single extruder, heated PEI buildplate and was unmodified from factory hardware. The CR-10S was outfitted with a 0.4 mm single extruder, heated glass buildplate and was unmodified from factory hardware. Adapters were initially printed in a variety of filaments including polylactic acid (PLA), polyethylene terephthalate glycol (PET-G) and thermoplastic polyurethane (TPU) to test for printability and usability. Non-PLA adapters were only printed during prototyping, but ultimately were not recommended or tested. PLA was chosen given its accessibility, ease of use and cost–effectiveness. Additionally, PLA has been shown to be biocompatible in many use cases [16].

Print settings were selected in order to minimize the need for infill and to ensure that the adapter had enough perimeters and walls to minimize the gaps between layers, gaps between walls and gaps in infill. Though PLA is not porous, the process of fused deposition modeling (FDM) 3D printing may allow for small porosity defects [17]. Minimizing these gaps by modifying print settings would allow for mitigation of penetration of liquids or air and allow for ease of sterilization [18]. Print settings shown in Table 1 were used. However, these may vary based on software and printer. For most printers, the default PLA settings will be sufficient.

**Table 1.** Outlines the print settings used in this design for a Taz5 Printer with eSun PLA + filament.

Select print setting	Value
Layer height	0.25 mm
Wall line count	4
Top/bottom layers	4
Infill density	20–50%
Printing temperature (nozzle)	205°C
Printing temperature (build plate)	60°C
Supports	None
Brim/raft	None
Print speed	60 mm/s
Print time	∼3 hours per adapter

### Usage & fit testing

Before being taken in for formal QLFT at The University of Kansas Health System (TUKHS), a water seal test was performed. The wearer donned the mask, our 3D printed adapter and viral filter combination and was submerged into water such that the water level was above the connection of the filter to the adapter, but below the top of the filter. This was done to allow for identification of leaks in the system. If the wearer felt water entering the mask or if air was seen escaping in the adapter fitment, the model was identified as not air or water-tight. This test was only performed before a version of the adapter was sent for QLFT at TUKHS.

At TUKHS, the design was fit tested by the facilities staff using a standard saccharin solution aerosol protocol following Occupational Safety and Health Administration guidelines [19]. Although QNFT was not done, successful QLFT is recommended by OSHA and has been shown to be predictive of successful QNFT [12,19]. At TUKHS, participants were those who were not able to pass fit testing with the standard N95 respirators available at TUKHS. None of the designers of the adapter or authors of this paper were present for fit testing, to ensure an unbiased fit test.

### Study design

IRB approval was not requested nor required for this study because this was essentially a proof of concept and quality improvement initiative. The design went through the proper channels at TUKHS for testing and approval of PPE during the height of the pandemic. Although further analysis and study to prove efficacy is required, the purpose of this study and article is to discuss the design, manufacturing and validation of the snorkel mask adapter concept.

## Results

Before being sent to TUKHS, the 3D-printed adapter along with snorkel mask and viral filter was water seal tested following the methods described above. After a few iterations of designs, no water was found to leak into the adapter or mask, thus ensuring that the seal between both mask and adapter as well as adapter and filter was secure. This was done as an initial validation test before sending the adapter for fit testing at TUKHS.

At TUKHS, 3832 people have been fit tested at least once for standard half face respirators. Of these, 211 people are currently listed as powered air purifying respirator (PAPR) only, placing a significant load on access to PAPR devices. The snorkel mask, adapter and viral filter combination was fit tested on 22 individuals of the 3832 people who required an N95 mask but were not able to pass QLFT with the masks available to them at the time. Three sizes of Ocean Reef Uno snorkel masks (along with 3D printed adapter and viral/bacterial filter) were used: S/M, M/L, L/XL. Out of the 22 individuals tested, all 22 of them were able to pass QLFT with the snorkel mask (one of three sizes), adapter, and Hudson RCI viral/bacterial filter combination. It was deemed appropriate that five of these 22 individuals were given snorkel masks, adapters and filters to use as necessary. These results show that the adapter, with viral/bacterial filter and appropriately sized snorkel mask, was able to pass fit testing on every individual who required it. As such, it was approved by TUKHS for individuals who needed it.

## Discussion

The results of the fit testing at TUKHS is promising for this N95 alternative. More extensive testing can and should be done, including QNFT. However, the initial results suggest that this mask could be efficacious at a larger scale. Previous studies show that of those who passed QLFT (n = 463) with all N95 FFR models, 86.9% (n = 459) also passed QNFT. This suggests that QLFT, although less rigorous than QNFT, does correlate highly with proper mask fitment [12].

Since the time of design and testing of the presented snorkel mask adapter, snorkel mask retailers and third-party companies alike have designed and tested their own injection molded snorkel mask adapters to be used with approved filters. Although these adapters may be less prone to the caveats of 3D printing [18], these products have only recently begun to be distributed world-wide. The lead time that is required for such large-scale production combined with complications of international distribution of the products leaves the supply chain with the same weaknesses that the world experienced not only in March of 2020 with the COVID-19 pandemic but also in 2009 H1N1 influenza pandemic and the 2014 Ebola virus epidemic [20]. In anticipation of future disturbances of the PPE supply chain (both traditional PPE and after-market adaptations such as a snorkel mask adapter), the need continues to exist for stopgap PPE that can be rapidly designed, produced and distributed locally using such technologies as 3D printing [21].

Although the Centers for Disease Control and Prevention (CDC) provides guidance to healthcare facilities on the optimization of the supply of N95 respirators during times of shortages [22], the research provided by the CDC acknowledges the increased risk and limitations associated with the strategies provided [23,24]. For example, the CDC cites the American Journal of Infection Control which states that a maximum of five consecutive donnings is the point at which the mask fit starts to suffer as a result of repeated use [24], and the Journal of Occupational and Environmental Hygiene which states that repetitive use of a single N95 mask increases the risk of self-inoculation and causes a decline of the mask's efficacy [23]. Some methods of sterilization for reuse can even decrease the filtration efficiency from the original nominal 95 to below 65%. Even repeated steam sterilization drops filtration efficiency after three treatments [25]. Accordingly, the CDC guidelines are still limited to the finite use of a resource which has been proven to be susceptible to supply chain disruption as discussed. This is precisely where the role of stopgap PPE comes into play as supply dwindles to a point where further reuse of PPE would be harmful to the user.

A unique feature of the design presented in this manuscript is the specificity of the design for the situation and supply levels of PPE in the Kansas City metropolitan area at the time of its design. Though bacterial/viral filters are also a finite resource, they were in relative abundance compared with N95 respirators and other PPE necessitated by the COVID-19 pandemic. A likely contributing factor for the relative abundance of the bacterial/viral filters is that their most common use is in surgical anesthesia. With the cessation of elective surgical procedures in the metropolitan area in the weeks following high infection rates in March of 2020, many ambulatory surgery centers and other noncritical care centers were temporarily closed. Although the anesthesia equipment used in these surgery centers were not intrinsically designed for use in infection control measures at the degree of filtration necessitated by COVID-19, adaptive components such as the design presented in this manuscript allowed for the maximization of available resources.

With the aforementioned weaknesses in the PPE supply chain withstanding, adaptive considerations such as the snorkel mask adapter design must continue to be pursued in cautious preparation for future challenges. Accordingly, International Organization for Standardization (ISO) guidelines were not taken into account at the time of designing. Rather, we produced an adapter that had proven adequate fitment with the brands of devices which were in relative abundance at the facilities with which we worked with in the design process. Further work could be done in order to ensure that these adapters meet the ISO standards (ISO-5356) for conical connectors [26].

Critical to this design is its adaptability. While the design presented here is designed to work with a specific brand of snorkel mask and bacterial/viral filter, it may be easily adapted and rapidly manufactured to work with components available to the healthcare organization in need of stopgap PPE. The authors cannot comment on this design's compatibility with other brands and types of snorkel masks or bacterial/viral filters outside of those specifically mentioned in the manuscript but encourage other makers to modify the design as needed. It has been well discussed that there are variations between STL and the product of 3D printing and slight inconsistency between prints [27]. Though these variances have shown to be statistically significant, the absolute differences are small enough to not affect the fitment of the adapter but is ultimately a tradeoff for the rapid availability provided by fused deposition modeling 3D printing [27].

Persistently increasing caseloads and PPE shortages necessitates an urgent dissemination of these preliminary results. No authors were present during the fit testing, to ensure unbiased testing. The authors do not advocate for this design as a replacement of traditional N95 masks or other PPE but do endorse this design as a stopgap measure, proven to be effective in situations of dire PPE shortage or for individuals who have failed fit testing with conventional PPE.

## Conclusion

The results of the fit testing at TUKHS is promising for this N95 alternative. More extensive testing can and should be done, including QNFT. Persistently increasing caseloads and PPE shortages necessitates an urgent dissemination of these preliminary results. The authors do not advocate for this design as a replacement of traditional N95 masks or other PPE but do endorse this design as a stopgap measure, proven to be effective in situations of dire PPE shortage or for individuals who have failed fit testing with conventional PPE.

**Summary points**

- The shortage of personal protective equipment (PPE) across the country has been widely discussed throughout the COVID-19 pandemic and unfortunately persists despite amidst increasing caseloads.
- Additionally, there have been reports of poor-fitting masks, a problem that is magnified by shortages.
- The lack of adequate access to conventional N95 masks pushed for some to pursue 3D printing and locally distributing their own manufactured masks as substitutes when PPE, including N95 masks, were not readily available.
- The design presented is an adapter that can be used with a commercially available snorkel mask in order to serve as a full face respirator in either the case of a PPE shortage or more pertinently for those who are unable to pass fit testing with the available N95 respirators at their respective facilities.
- The design for the snorkel mask adapter was generated using commercial CAD software and exported to an STL file for compatibility with hobbyist 3D printer workflows.
  - Two combinations of slicer and 3D printer were tested: the Cura for LulzBot slicer (version 3.6.18) was used to prepare G-code for a Lulzbot Taz5, and PrusaSlicer (version 2.3) was used to prepare G-code for a Creality CR-10S. The Lulbot Taz5 was outfitted with a Lulzbot 0.5 mm single extruder, heated PEI buildplate and was unmodified from factory hardware. The CR-10S was outfitted with a 0.4 mm single extruder, heated glass buildplate and was unmodified from factory hardware.
  - Polylactic acid (PLA) was selected for its ease of use, cost–effectiveness and biocompatibility.
- Assembled masks were fit tested by qualitative fit testing (QLFT) at The University of Kansas Health System (TUKHS) in Kansas City, KS on 22 individuals who required an N95 mask but were not able to pass QLFT with the masks available to them at the time. Out of the 22 tested, all 22 of them were able to pass QLFT with the snorkel mask, adapter and viral/bacterial filter combination.
- Although PPE has improved since the origin of this design and some snorkel mask adapters are now commercially available, the need continues to exist for stopgap PPE that can be rapidly designed, produced and distributed locally using such technologies as 3D printing.
  - Lead time and complications of international distribution leaves the supply chain with the same weaknesses that the world experienced not only in March of 2020 but also in previous times of PPE shortages.
  - Reuse of respirators increases the risk of self-inoculation and reduces the mask's efficacy and filtration.
- While the design presented here is designed to work with a specific brand of snorkel mask and bacterial/viral filter, it may be easily adapted and rapidly manufactured to work with components available to the healthcare organization in need of stopgap PPE.
- The authors do not advocate for this design as a replacement of traditional N95 masks or other PPE but do endorse this design as a stopgap measure, proven to be effective in situations of dire PPE shortage or for individuals who have failed fit testing with conventional PPE.
